# COVID-19 pandemic: a viewpoint from Asia

**DOI:** 10.1186/s42269-020-00337-5

**Published:** 2020-05-29

**Authors:** Md. Abdullah-Al-Shafi

**Affiliations:** Institute of Information Technology (IIT), University of Dhaka, Dhaka, Bangladesh

**Keywords:** COVID-19, Coronavirus, SARSCoV-2, Severe acute respiratory syndrome, Asia

## Abstract

The expansion of severe acute respiratory syndrome (SARS) coronavirus 2 (SARSCoV-2) has now procured on epidemic percentages, affecting more than 190 nations in a matter of weeks. A widespread SARSCoV-2 contagion begun in Wuhan, Hubei Province, China, and circulate through China and beyond in December 2019. The containment events in China have lessened new instances by more than 90%, but this diminution is not the case to a different place. European countries like Italy and Spain have been the most affected. In Asia, the COVID-19 brings a catastrophe where after China mainland, countries like Iran and South Korea have been affected. There is now severe apprehension concerning the Asian health care system’s ability to effectually counter to the necessities of patients who are infected and need rigorous precaution for COVID-19. The patient’s ratio in special care reported in Iran has perpetually been between 6 and 8% who are rapidly infected. As about South Korea, the ratio is 3% and 4% who are rapidly infected. This analysis emphasizes the epidemiology of COVID-19, its effects in the Asia continent, and active case study of COVID-19 including the distinct countries.

## Introduction

The wave of SARSCoV-2 arisen in December 2019 and by this time turn up the required epidemiological measures for it to be announced a pandemic. So far this contagion affected more than 500,000 people in 190 nations; hence, an organized worldwide response is frantically required to concoct physical monitoring to meet this exclusive confront (Remuzzi & Remuzzi, 2020). The active case exposure rate is fluctuating daily and can be followed in about real time on the dashboard supported by Johns Hopkins University (Coronavirus 2019-nCoV, 2020). Moreover, some other sites provide regular statistics regarding COVID-19 occurrence worldwide (Coronavirus Cases, 2020). As the middle of February 2020, an Asian country, China, carries the outsized encumbrance of disease and death, although the frequency in other Asian countries, in North America, and Europe persist minimal so far. Since March 22, 2020, at least one active situation of COVID-19 has been affirmed in each country in Asia. The focal point of the epidemic in Asia is in China and Iran. Nations that have been inopportune enough to have been revealed to this disease already have, inconsistently, precise lessons to convey, though the containment degrees implemented in China have lessened new cases in almost 90% while this diminution is not the circumstance in other countries, including Iran, Spain, and Italy (Remuzzi & Remuzzi, 2020). Asian countries including China reported more than 80,000 cases, Iran has had more than 40,000 authorized active cases, and South Korea testified almost 10,000 as per (Coronavirus Cases, 2020). Till March 30, 2020, Italy has listed more demises because of the COVID-19 epidemic and over two-thirds of these reported patients had cardiovascular syndromes, diabetes, or other related diseases. Total of 170,365 active cases has been authorized so far in Asia till March 30, 2020, where china confirmed more than 81,400 cases that put china the maximum infected area in Asia (Coronavirus Cases, 2020). Iran is the second infected country holding more than 41,400 cases (Coronavirus Cases, 2020). Turkey has reached the functional cases in 10,800 (Coronavirus Cases, 2020), and South Korea reached the number almost 10,000 (Coronavirus Cases, 2020). Other Asian countries’ active cases are inclining in time. Moreover, intensifying COVID-19 catastrophe pressures to apprehensively hit developing states, not only as a health emergency in the short period but as an overwhelming business and social problem over the whiles and years to come.

**Fig. 1:**
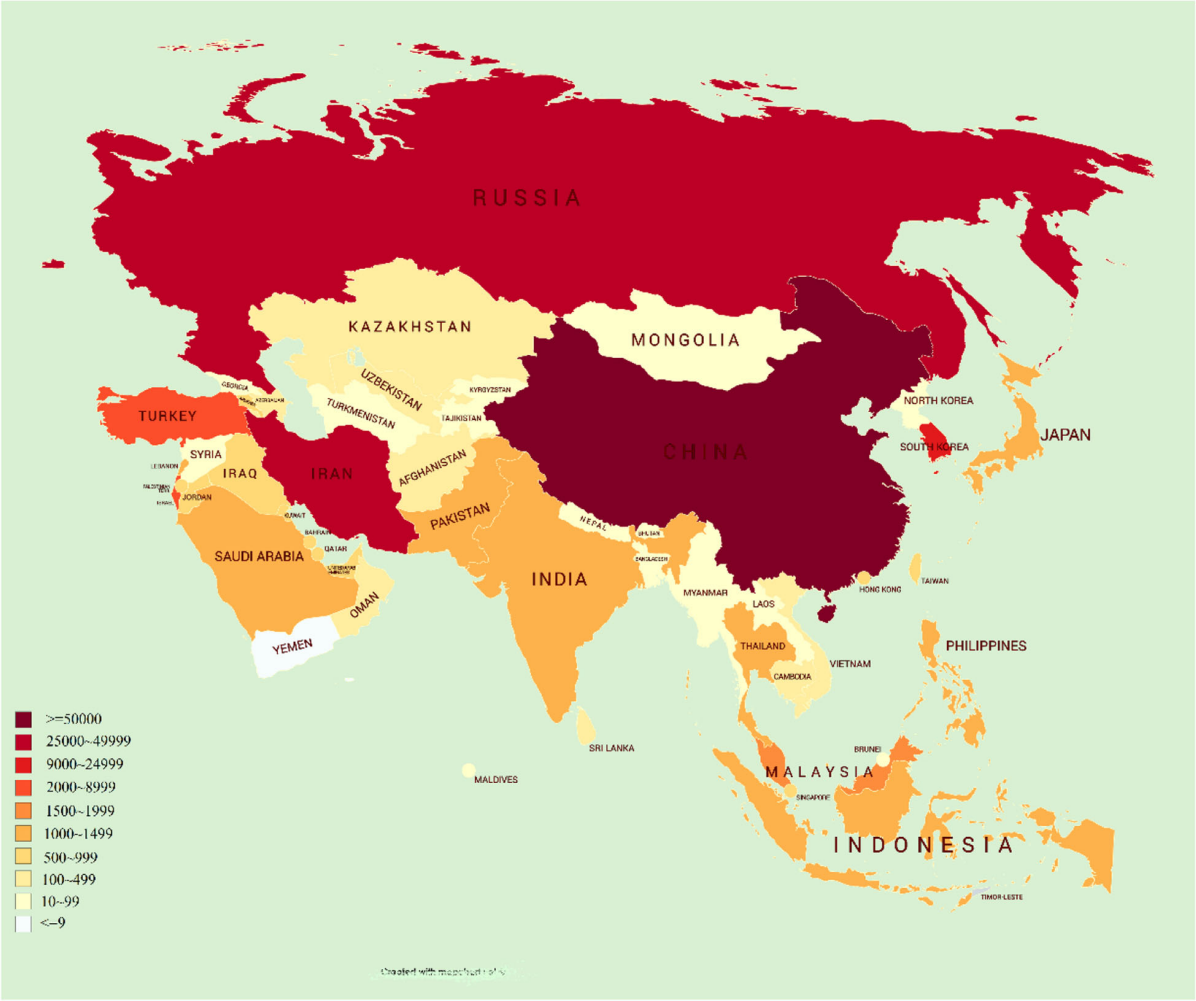
Asian countries reported confirmed active cases of COVID-19, March 30, 2020

Revenue defeats are expected to surpass $220 billion in rising countries (UNDP, 2020) and an assessed 55% of the worldwide population holding no access to community protection. Hence, forfeitures will resonate throughout cultures, human justices and, in the harshest cases, necessary food security and nourishment. Most of the nations in Asia region are developing phase and there is a strong motive that food and nutrition will be a vulnerable outlook for developing countries. World Health Organization (WHO) is assisting developing countries to set up for, respond to, and recuperate from the COVID-19 contagion, centering acutely on the most susceptible (World Health Organization, 2020a). Up to now, 14 Asian countries reached a thousand active cases and another 10 countries are reaching a thousand cases, indicated in Fig. 1.

## COVID-19 etiology

The early experimental sign of the SARSCoV-2-related disease COVID-19 which legalized case detection was pneumonia but more contemporary reports also explain asymptomatic infections and gastrointestinal signs, particularly among young children (Chan et al., 2020). In initial analysis, the overall viral genome study discloses that the virus assigns 88% succession identity to two bat-originated SARS-like coronaviruses, nonetheless more detached from severe acute respiratory syndrome coronavirus (SARSCoV) (Lu et al., 2020). Consequently, it was provisionally known as 2019-novel coronavirus. Coronavirus is an encircled and unique stranded ribonucleic acid termed for its solar corona as appearance caused by 9–12 nm extended surface prickles (Zu et al., 2020). Four significant proteins’ structure coded by the coronaviral genome on the wrap, one of which is the prickles, protein that fixes to angiotensin-converting enzyme-2 receptor and referees’ successive synthesis concerning the wrap and host cell membranes to assist viral access into the host cell (Xu et al., 2020a). Coronavirus Study Group of the International Committee on Taxonomy of Viruses (ICTV) decisively listed it as SARSCoV-2 focused on classification, phylogeny, and traditional practice (Gorbalenya, 2020). Following, WHO classified the syndrome began by this coronavirus as coronavirus disease 2019 (COVID-19) (World Health Organization, 2020b). On the base of existing statistics, it appears that COVID-19 may be primarily hosted by bats, then may conveyed to persons via pangolin (Lam et al., 2020) or other wild creatures (Lu et al., 2020) but consequent proliferated through person to person transmission.

## COVID-19 effect on Asian countries

**Fig. 2:**
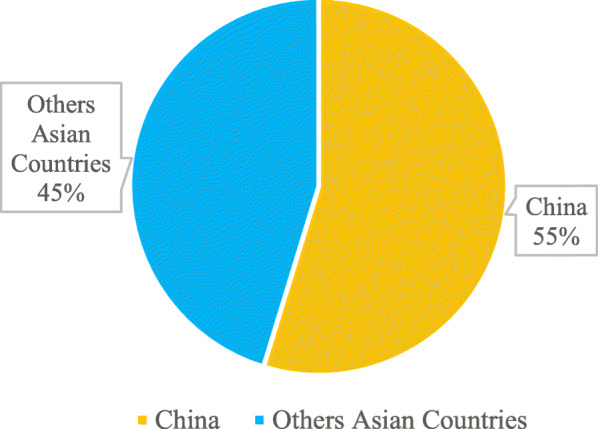
COVID-19 cases in Asian countries

The enduring COVID-19 epidemic affects the Asian countries mostly the developing economies in Asia within frequent means, entering reduce tourism, business tour, supply breaks, intercountry production, internal demand, and health effects. The significance of the economic bearing will differ on how the rash changes that endures extremely tentative. Instead of concentrating on a particular estimation, it is imperative to study a series of circumstances, measure the effect provisional on these circumstances appearing, and to revise the situations as required. The series of situations studied in this brief submit an inclusive effect of 0.1 to 0.4% of global gross domestic product (GDP) or $77 billion to $347 billion (Abiad et al., 2020), through a relative case estimate of $156 billion or 0.2% of worldwide GDP. Almost two-thirds of the effect falls on China, where the epidemic has been concerted to date, as showed in Fig. 2.

**Fig. 3:**
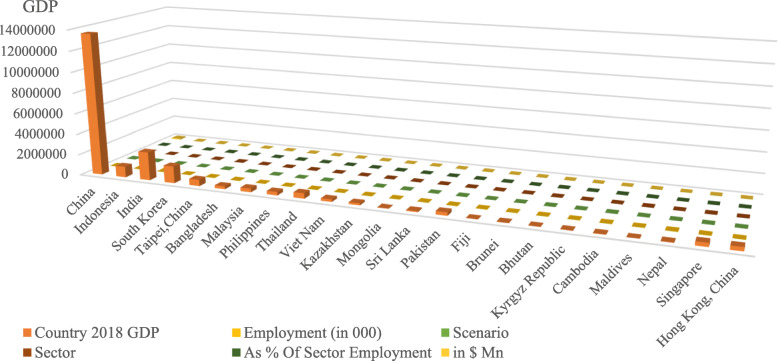
Moderate case impact of COVID-19 rash on developing Asian economies

The projected effect on distinct developing Asian countries and on sectors inside these economies is presented in Fig. 3, including a hypothetical moderate case situation. In these circumstances, where cautionary manners and limitations such as journey bans start enabling 3 months after the epidemic extended and limitations were inflicted in late January, inclusive forfeitures could spread $156 billion, or 0.2% of worldwide GDP (Abiad et al., 2020). China would report for $103 billion of those forfeitures or 0.8% of its GDP. The remaining of emerging Asia would forfeit $22 billion, or 0.2% of its GDP.

Asian Development Bank proposes a report (Abiad et al., 2020) that can assist Asian governments as they express vibrant and pivotal feedbacks to lessen the social and financial collisions of this outbreak. The entire GDP of a country is fluctuating due to this rash mostly for developing countries, for instance, tourism and commercial travel. Tourism is a notable resource of expenses for many developing economy countries in Asia. Worldwide tourism grosses credit for more than 40% of the GDP in economies like Maldives and Palau, and entire travel and tourism surpasses 10% of GDP in nearly half of Asian Development Bank (ADB)’s associates (Wu et al., 2020). It is apparent that these sites are anticipated to deteriorate, on account of frequent travel prohibitions with cautionary behavior. A recent substantial travel prohibition is the one obliged by China itself. On January 24, 2020, the Chinese government obliged a travel prohibition on all outbound tourism by travel companies (Chang et al., 2020).

**Fig. 4:**
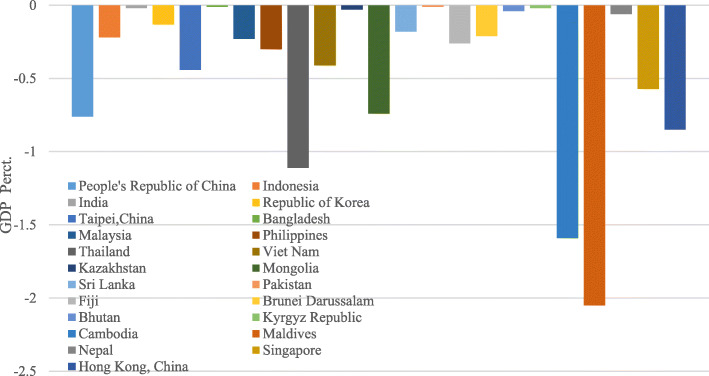
The economic impact of the COVID-19 outbreak on developing Asia (March 2020)

In Asia, many developing countries depend on China in various businesses. China is a foremost trade market for many ADB developing countries, with trades to China being a significant portion of GDP. Thus, a fall in the requirement for imports and exports from China is possible to be felt broadly. The GDP influences in quite decisive for ADB member countries as denoted in Fig. 4.

There have been extensive manufacture interruptions because of enforced industry closings and the employees’ inability to move to work, along with troubles to business and commerce due to frontier closes, travel forbids, and other limitations on the transfer of products, persons, and capital. High-frequency pointers propose that manufacture in the developing countries as a whole may reduce to 50% of regular levels nevertheless is now stabilizing. The socioeconomic sophisticated China, Russia, Japan, South Korea, Turkey, Taiwan, Saudi Arabia, Qatar, Bahrain, UAE, Kuwait, and Brunei, almost all other developing countries might be harshly spoiled before any applicable remedy attains. Alongside the World Bank assigning $12 billion and the International Monetary Fund (IMF) $50 billion (Djalante et al., 2020), nations must neither be dazzled about the profundities the syndrome could still spread, nor the duration of the recuperation. The infection is Asia’s awaken call to sincerely regulate emergency policies on every potential front.

## Prospective clarification

**Fig. 5:**
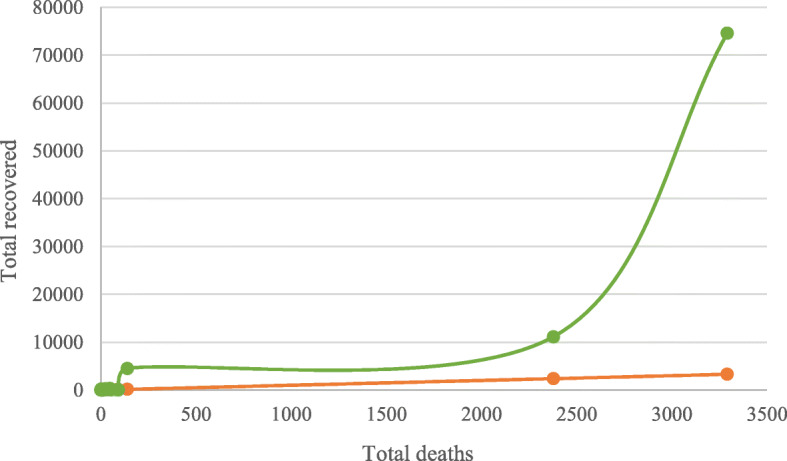
Total recovered vs death in COVID-19 (March 30, 2020)

Currently, COVID-19 appears to spread from human to human through the uniform process as usual cold or flu viruses, i.e., face interaction with a sneeze, or from interaction with secretions of humans who are diseased. The function of fecal oral interaction is however, to be resolved in COVID-19 although it was found to appear through the SARS epidemic (Chinese SARS Molecular Epidemiology Consortium, 2004). The isolation of Wuhan City looks to have reduced the worldwide extent of COVID-19, yet the consequence is anticipated to be ephemeral. To date, enormous people are affected globally in COVID-19 and many of them are recovering but a number of demises become cumulative in time as shown in Fig. 5.

**Fig. 6:**
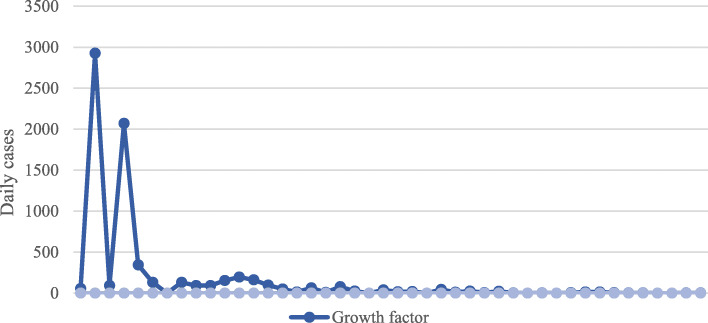
Growth factor in COVID-19 (March 30, 2020)

Attempts are presently proceeding in China, almost 190 nations to which diseased persons have toured, and in communal transportations, for instance, voyage ships, to disrupt communication of all present and possible chains of transmission, through eradication of COVID-19 in humanoid inhabitants as the conclusive goal. Though the number of global active cases is inclining with time, for instance, the number of actives cases in Iran on March 29, 2020 was 39, 875 but after 1 day on March 30, 2020, the number increases to 41,495 (Coronavirus Cases, 2020). In Turkey, the number was 10,827 on March 29, 2020 and later it was 13, 531 on March 30, 2020 (Coronavirus Cases, 2020). Similar active case can be found for Russia, South Korea, Japan, Saudi Arabia, and other Asian country. The growth factor for active cases is surging for all Asian countries as illustrated in Fig. 6, though China has taken several strict verdicts to put a chain on COVID-19 and seems they are attaining some sound effect (Homeland Security Today News, 2020).

Decisively, another key objective of China’s present epidemic response actions is to facilitate “buy time” for science to overtake before COVID-19 enhances too pervasive. Though, the progress is not succeeded in many cases as most of the countries worldwide are facing disaster for COVID-19. China should turn on regulating strategies and policies as new evidence becomes accessible (Cowling & Leung, 2020). China is very appreciative of the assistance it is obtaining from the worldwide technical, health, and social communities. WHO proposed some strategic points for COVID-19 (World Health Organization, 2020c), for instance, interrupt person-to-person communication including demoting secondary contagions among adjacent contacts and health care personnel and avoiding transmission expansion occasions; immediate classify, separate, and supervision for patients; identify and diminish conduction from the animal source; connect precarious danger and consequence evidence to all communities and counter misrepresentation, etc.

A strong community is building up to prevent this rash, though the scientist or experts have not enough evidence regarding this disease. To address, a number of concerns of infection monitor, evidence flow, resuscitation guidance, sophisticated intensive care unit (ICU), and psychological comfort of workforce, some devised frequent standards and explanations, which we expect to assist other ICUs formulate for COVID-19 (Wax & Christian, 2020). A compulsive results regarding acute respiratory syndrome of COVID-19 is presented in (Xu et al., 2020b) that may be a way to cope with the disease. A group of researchers in Bangladesh has formed a $3 analysis kit that they appeal can perceive COVID-19 in within 15 min (Medical device network, 2020). The Directorate General of Drug Management gave its approval for the bulk manufacture of the kit. The physiognomies of COVID-19 are still researched (Tian et al., 2020) and optimistically nations will get clarification of this disaster.

Maximum developing economies in Asia are now acting to the COVID-19 epidemic in several ways. Many managements have assembled inter-bureau task forces and other directing processes to confirm a synchronized response. To defend their inhabitants, several developing members of ADB have executed numerous forms of voyage restrictions, reinforced screening processes and quarantine guidelines, and commenced deportation of their citizens from epidemic pretentious economies. Economies also supporting their health regularities by employing contact tracking when required, confirming satisfactory provisions of personal protective apparatus, supporting research laboratory facilities, and confirming passable interaction of threats. Significantly, many nations by now are commencing compassionate macroeconomic schemes. Many nations have excise interest ratios, persisting a rotation of easing that initiated in 2019, and others are also locating in place accommodating fiscal measures (Abiad et al., 2020). ADB is supporting its associates in responding to the COVID-19 epidemic throughout business, education, and partnerships. ADB assists on the funding side includes a sanctioned $2 million specialized assistance allowance to aid the China and the Greater Mekong Subregion to identify, prevent, and act to the enduring COVID-19 epidemic and future infectious disease outbursts, and a $2 million regional specialized assistance allowance for all developing countries to sustain response undertakings in the region (Abiad et al., 2020). A reformation of existing properties is also taking the position, as ADB has a number of health development projects in the territory totaling $469 million and a few of this can be transferred in response to the epidemic.

## Conclusions

Practical psychoanalysis and financing in public health structure with capacity are decisive to efficiently counter to outbreaks like COVID-19. It is perilous to endure to progress worldwide observation, cooperation, synchronization, and communication regarding this notable rash and to be even better ready to counter to upcoming novel public health menaces. The epidemic starts in China but now spreads throughout the world and for Asia, and systematized and prepared health practice can diminish people to get more infected. Lastly, intense research is imperative to recognize the root of the epidemic by analysis of faunas and animal handlers in marketplaces to specify evidence required for the preclusion of future coronavirus epidemics.

## Data Availability

Not applicable.

## Abbreviations

ADB: Asian Development Bank
COVID-19: Coronavirus disease 2019
GDP: Gross domestic product
ICTV: International Committee on Taxonomy of Viruses
ICU: Intensive care unit
IMF: International Monetary Fund
SARS: Severe acute respiratory syndrome
SARSCoV: Severe acute respiratory syndrome coronavirus
SARSCoV-2: Severe acute respiratory syndrome coronavirus 2
WHO: World Health Organization
